# Duration of cold exposure defines the rate of reactivation of a perennial *FLC* orthologue via H3K27me3 accumulation

**DOI:** 10.1038/s41598-020-72566-7

**Published:** 2020-09-29

**Authors:** Haruki Nishio, Koji Iwayama, Hiroshi Kudoh

**Affiliations:** 1 Center for Ecological Research, Kyoto University, 2-509-3 Hirano, Otsu, 520-2113 Japan; 2 Faculty of Data Science, Shiga University, 1-1-1 Bamba, Hikone, Shiga 522-8522 Japan; 3 PRESTO, Japan Science and Technology Agency, Kawaguchi, 332-0012 Japan

**Keywords:** Plant genetics, Epigenetics

## Abstract

Vernalisation is the process in which long-term cold exposure makes plants competent to flower. In vernalisation of *Arabidopsis thaliana*, a floral repressor, *AtFLC*, undergoes epigenetic silencing. Although the silencing of *AtFLC* is maintained under warm conditions after a sufficient duration of cold, *FLC* orthologues are reactivated under the same conditions in perennial plants, such as *A. halleri*. In contrast to the abundant knowledge on cold requirements in *AtFLC* silencing, it has remained unknown how cold duration affects the reactivation of perennial *FLC*. Here, we analysed the dynamics of *A. halleri FLC* (*AhgFLC*) mRNA, H3K4me3, and H3K27me3 over 8 weeks and 14 weeks cold followed by warm conditions. We showed that the minimum levels of *AhgFLC* mRNA and H3K4me3 were similar between 8 and 14 weeks vernalisation; however, the maximum level of H3K27me3 was higher after 14 weeks than after 8 weeks vernalisation. Combined with mathematical modelling, we showed that H3K27me3 prevents a rapid increase in *AhgFLC* expression in response to warm temperatures after vernalisation, which controls *AhgFT* expression and the initiation of flowering. Thus, the duration of cold defines the rate of *AhgFLC* reactivation via the buffering function of H3K27me3 against temperature increase.

## Introduction

Vernalisation, a major flowering pathway, is the process that makes plants competent to flower following long-term cold exposure^1,2^. Studies investigating the natural variation in vernalisation requirement have identified one of the main responsible genes, *FLOWERING LOCUS C* (*FLC*) in *Arabidopsis thaliana* (hereafter *AtFLC*)^3,4^. Molecular genetic studies have revealed that *AtFLC*, which encodes a MADS-box protein, represses flowering before vernalisation in winter annual ecotypes^5,6^. *AtFLC* suppresses flowering, at least in part, by repressing the expression of the floral activators *AtFT*, *AtFD*, and *AtSOC1*^7,8^.

In the vernalisation process of *A. thaliana*, *AtFLC* undergoes quantitative silencing, which depends on the duration of cold^9^. This process is accompanied by the decrease in active histone modifications, especially histone H3 lysine 4 trimethylation (H3K4me3), at a region around the transcription start site known as the nucleation region, and the increase of a repressive histone modification, histone H3 lysine 27 trimethylation (H3K27me3), at the same region^10–16^. After return to warm conditions, H3K27me3 spreads to the entire gene locus and silencing is maintained^12–16^, which induces the expression of floral activators and flowering under favourable long-day conditions^7,9^. The duration of cold required for full vernalisation is different between *A. thaliana* accessions^17–19^. Accessions from Sweden require a longer duration of cold for stable *AtFLC* repression compared with accessions from other countries, and variation in the vernalisation response is correlated with epigenetic silencing of *AtFLC*^17–19^. In Col *FRI*^Sf2^, the H3K27me3 level at the nucleation region of *AtFLC* was found to be saturated after 6 weeks of cold^15^. In Lov-1, which was collected from northern Sweden, H3K27me3 accumulation at *AtFLC* was delayed and a longer duration of cold was required to reach H3K27me3 levels equivalent to those in Col *FRI*^Sf2^^18,19^.

In contrast to the abundant knowledge on cold requirements in the *AtFLC* silencing process, the effect of cold duration on the upregulation of perennial *FLC* orthologues on return to warm has remained to be determined. *FLC* orthologues are silenced in a quantitative manner under cold conditions as well as *A. thaliana*; however, they are upregulated under warm conditions following vernalisation in perennial plants, *Arabidopsis halleri*^20–25^, *Arabidopsis lyrata*^26^, and *Arabis alpina*^27–29^. H3K27me3 accumulates at perennial *FLC* orthologues under cold conditions and is removed from the locus in response to warm temperatures^24,25,27^. In perennial plants, the upregulation of *FLC* orthologues under warm conditions following vernalisation would antagonise the upregulation of *FT* orthologues and flowering. Thus, it is expected that an insufficient cold duration would affect flowering through the perturbation of perennial *FLC* upregulation.

A mixed approach, including time series data and mathematical modelling, has been used to investigate the molecular mechanisms underlying the vernalisation process^14,20,21,24,30–33^. We previously reported the seasonal dynamics of *AhgFLC* mRNA, H3K4me3, and H3K27me3 over 2 years in a natural population, and constructed mathematical models to analyse the properties of the regulatory system^24^. Numerical simulations revealed that bidirectional interactions between H3K27me3 and H3K4me3 are required for *AhgFLC* expression to respond to long-term temperature trends in a fluctuating natural environment^24^.

In this study, we analysed the dynamics of *AhgFLC* mRNA, H3K4me3, and H3K27me3 over 8 weeks and 14 weeks of cold conditions followed by warm conditions (referred to as 8 weeks and 14 weeks vernalisation treatments, respectively), and then performed numerical simulations to determine the role of H3K27me3 in the *AhgFLC* regulation over the two conditions. We showed that the minimum levels of *AhgFLC* mRNA and H3K4me3 were similar between the 8 weeks and 14 weeks vernalisation treatments, while the maximum level of H3K27me3 was higher in the 14 weeks than in the 8 weeks vernalisation treatment. Combined with mathematical modelling, we showed that H3K27me3 plays a key role in moderating the rate of *AhgFLC* reactivation and controls the initiation of flowering.

## Results

### Dynamics of *AhgFLC* mRNA and histone modifications in the vernalisation treatments

**Figure 1:**
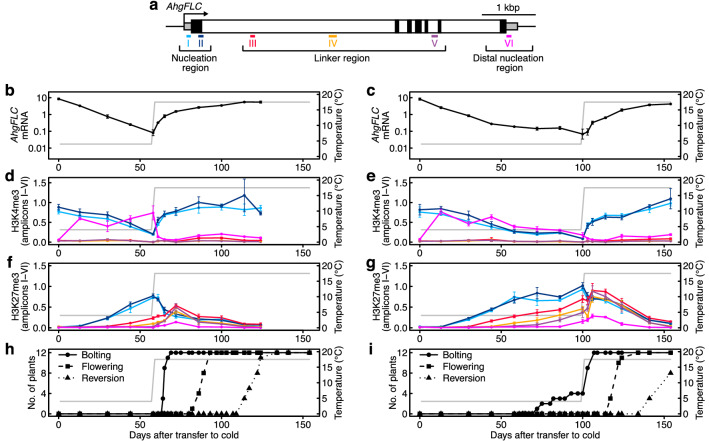
Dynamics of *AhgFLC* mRNA and histone modification levels in the vernalisation treatments. (**a**) Structure of the *AhgFLC* locus with untranslated regions (grey), exons (black), and introns (white); distribution of six H3K4me3 and H3K27me3 ChIP amplicons in different colours and the definitions of the nucleation, linker, and distal nucleation regions. (**b**–**g**) The dynamics of *AhgFLC* mRNA (**b**, **c**), H3K4me3 at amplicons I–VI (**d**, **e**), and H3K27me3 at amplicons I–VI (**f**, **g**) in the 8 weeks (**b**, **d**, **f**) and 14 weeks (**c**, **e**, **g**) vernalisation treatments. (**h**, **i**) A time course of reproductive transition (bolting, flowering and reversion) of the study plants (see Methods for the definition of the stages) in the 8 weeks (**h**) and 14 weeks (**i**) vernalisation treatments. Reversion, leaf formation at the reproductive shoot apical meristem. In (**b**–**i**), temperature regimes are represented by grey lines (4 °C in cold and 20/15 °C D/N in warm, shown by the average value). The colour code in (**d**–**g**) corresponds to that in (**a**). In (**b**–**g**), the means and standard errors of biological replicates are shown. *n* = 2–4 (average, 3.3) and 1–4 (average, 3.4) for mRNA in the 8wk and 14 weeks vernalisation treatments, respectively. *n* = 4 for H3K4me3 and H3K27me3 at all amplicons in both vernalisation treatments. For each replicate, a pool of leaves from three plants (out of 12 plants) was analysed. The qPCR data are represented relative to *AhgACT2* (mRNA and H3K4me3) and *AhgSTM* (H3K27me3).

We analysed *AhgFLC* mRNA levels, and the H3K4me3 and H3K27me3 levels at six amplicons along the locus using a chromatin immunoprecipitation (ChIP), in the 8 weeks and 14 weeks vernalisation treatments (Fig. 1). As previously defined^24^, the *AhgFLC* locus was classified into three regions: the nucleation region, the linker region, and the distal nucleation region (Fig. 1a). In the 8 weeks vernalisation treatment, *AhgFLC* mRNA level continued to decrease under the cold condition (Fig. 1b). In the 14 weeks vernalisation treatment, the minimum *AhgFLC* mRNA level was observed after 8 weeks of cold, and was maintained up to 14 weeks of cold (99% decrease in mRNA during the first 8 weeks of cold; Fig. 1c). *AhgFLC* mRNA levels increased following transfer to warm conditions and returned to pre-vernalisation levels after 8 weeks of warm in both the 8 weeks and 14 weeks vernalisation treatments (Fig. 1b, c). The H3K4me3 levels at the nucleation region exhibited similar dynamics to the mRNA levels (Fig. 1d, e). In both the 8 weeks and 14 weeks vernalisation treatments, the H3K4me3 levels at the distal nucleation region, an indicator of the activity of antisense transcripts, *AhgCOOLAIR*^24^, increased under cold conditions, reaching the maximum level after 2 weeks of cold (Fig. 1d, e). The H3K4me3 level at this region decreased rapidly following transfer to the warm condition in the 8wk vernalisation treatment (Fig. 1d), whereas it decreased gradually to the end of cold treatment in the 14wk vernalisation treatment (Fig. 1e). Although the distal peak of H3K4me3 correlated with *COOLAIR* transcription in *A. thaliana*^15^, *AhgCOOLAIR* dynamics should be analysed to evaluate the correlation in *A. halleri*. In the 8 weeks vernalisation treatment, the H3K27me3 levels at the nucleation region continued to increase under the cold condition, decreased following transfer to the warm condition, and returned to the pre-vernalisation levels after 8 weeks of warm (Fig. 1f). The H3K27me3 levels at the linker region and the distal nucleation region increased slightly under cold, reaching the highest levels (within data points in the 8 weeks vernalisation treatment) 2 weeks after transfer to the warm condition, after which they then decreased (Fig. 1f). Thus, there was a lag of 2 weeks in the timing of the H3K27me3 peaks between the nucleation region and the linker/distal nucleation regions (Fig. 1f). In the 14 weeks vernalisation treatment, the H3K27me3 levels at all three regions reached higher levels than those in the 8 weeks vernalisation treatment (Fig. 1g). There was a lag of 1 weeks in the timing of the H3K27me3 peaks between the nucleation region and the linker/ distal nucleation regions in the 14 weeks vernalisation treatment (Fig. 1g). Similar patterns were observed using a second set of validated^34^ reference genes (Supplementary Fig. S1). Consistent with the function of *AhgFLC* as a floral repressor, bolting started 1 weeks after transfer to the warm condition, followed by flowering and reversion to vegetative growth in the 8 weeks vernalisation treatment (Fig. 1h). In the 14 weeks vernalisation treatment, bolting started after 10 weeks of cold, while flowering and reversion started only after transfer to the warm condition (Fig. 1i). Taken together, these data indicate that *AhgFLC* mRNA levels and the H3K4me3 levels at the nucleation region were saturated after 8 weeks of cold, and the minimum levels were similar between the 8 weeks and 14 weeks vernalisation treatments. However, the maximum level of H3K27me3 differed between the two treatments.

### Comparison of the influence of cold on flowering time between the 8 weeks and 14 weeks vernalisation treatments

**Figure 2:**
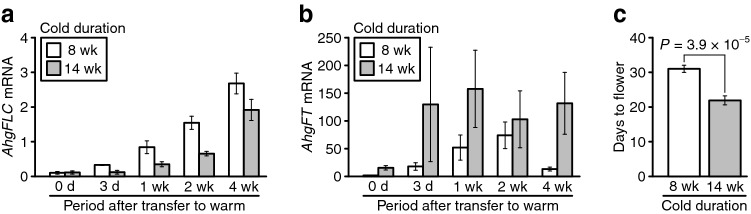
Differences in cold duration influenced the expression of flowering genes and flowering time. (**a**, **b**) Effects of cold duration (8 and 14 weeks) and period after transfer to warm conditions on the expression of *AhgFLC* (**a**) and *AhgFT* (**b**) mRNA. (**c**) Days to flower under warm conditions after 8 and 14 weeks vernalisation. In (**a**–**c**), the means and standard errors of biological replicates are shown. *n* = 2–4 (average, 3.4) and 4 for mRNA in the 8 weeks and 14 weeks vernalisation treatments, respectively. *n* = 12 for flowering time in both vernalisation experiments. In (**a**, **b**), a pool of leaves from three plants (out of 12 plants) was analysed for each replicate. The qPCR data are represented relative to *AhgACT2*.

Next, we compared *AhgFLC* mRNA levels after transfer to warm conditions between the 8 weeks and 14 weeks vernalisation treatments. *AhgFLC* mRNA levels were lower in the 14 weeks than in the 8 weeks vernalisation treatment after transfer to warm conditions (*P* = 0.00041, two-way ANOVA; Fig. 2a, Supplementary Fig. S2, and Table 1). Consistent with the function of *AhgFLC* as a floral repressor^20,21,24^, *AhgFT* mRNA levels were higher in the 14 weeks than in the 8 weeks vernalisation treatment after transfer to warm conditions (*P* = 0.026, two-way ANOVA; Fig. 2b, Supplementary Fig. S2, and Table 2). Consistent with these results, plants flowered earlier after 14 weeks compared with 8 weeks vernalisation (*P* = 3.9 × 10^−5^, Wilcoxon rank sum test; Fig. 2c). Therefore, a longer duration of cold delayed *AhgFLC* upregulation after transfer to warm conditions and advanced flowering time.

**Table 1:** The two-way ANOVA table for the *AhgFLC* expression.

Source	*df*	Sum Sq	Mean Sq	F value	P value
Cold duration	1	2.04	2.04	15.7	0.00041
Period after transfer to warm	4	23.9	5.99	46.1	1.3 × 10^−12^
Residuals	31	4.03	0.130

**Table 2:** The two-way ANOVA table for the *AhgFT* expression.

Source	*df*	Sum Sq	Mean Sq	F value	P value
Cold duration	1	50,243	50,243	5.51	0.026
Period after transfer to warm	4	42,041	10,510	1.15	0.35
Residuals	31	282,852	9124		

We observed large differences in *AhgFT* mRNA levels between replicates in the 14 weeks vernalisation treatment after transfer to warm conditions: replicate 4 showed higher levels than other replicates (Supplementary Fig. S3). All three individuals pooled for replicate 4 of 14 weeks vernalisation treatment had started bolting during cold exposure earlier than other replicates, whereas days to flower were similar across all replicates (Supplementary Fig. S3). Thus, the higher *AhgFT* mRNA levels in replicate 4 after transfer to warm did not necessarily correlate to flowering time. We transplanted naturally growing plants to laboratory conditions and their ages and genotypes were diverse, which may partly explain observed large differences in *AhgFT* mRNA levels between replicates. We speculate that vernalisation requirement is mostly met after 14 weeks of cold because some plants started bolting even during cold exposure (Supplementary Fig. S3).

### Mathematical modelling of *AhgFLC* mRNA levels

**Figure 3:**
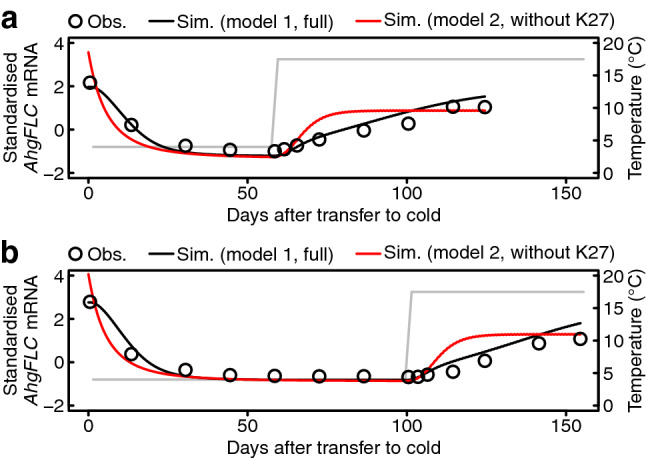
Comparison of *AhgFLC* mRNA dynamics between the mathematical models with and without the effect of H3K27me3. (**a**) Simulated *AhgFLC* mRNA levels in the 8 weeks vernalisation treatment are compared between models with (black line) and without (red line) H3K27me3, and are shown with the observed values (open circles). (**b**) Simulated *AhgFLC* mRNA levels in the 14 weeks vernalisation treatment. Otherwise the same as in (**a**). The observed values are shown as the means of biological replicates. Temperature regimes are represented by grey lines (4 °C in cold and 20/15 °C D/N in warm, shown by the average value).

To further evaluate the effect of H3K27me3 on *AhgFLC* mRNA dynamics, we performed numerical simulations using the differential equation models. We compared the full model that includes H3K27me3 (model 1) which was established to predict the mRNA and histone modification dynamics in a natural population of *A*. *halleri*^24^ and the model that lacks H3K27me3 (model 2). In model 1, H3K27me3 and H3K4me3 were assumed to antagonise each other at the two nucleation regions of *AhgFLC*, which was represented by differential equations. The mRNA level was modelled by linear regression with the H3K4me3 level at the nucleation region. The assumptions in model 2 is the same as those in model 1 except that all variables and parameters regarding the H3K27me3 states were excluded from the full model (see the Methods section for more detail). We optimised the parameters in the two models to fit the model outputs to the observed data. In model 1, the simulated H3K4me3 and H3K27me3 levels at the nucleation region—which are directly linked to the mRNA level—were highly correlated with the observed data (Spearman’s correlation coefficient *r* > 0.9; Supplementary Fig. S4), although correlations were lower at the distal nucleation region (Spearman’s correlation coefficient *r* = 0.65 and 0.84, respectively; Supplementary Fig. S4). The simulated *AhgFLC* mRNA levels in model 1 agreed well with the observed data in both the 8 weeks (Fig. 3a) and 14 weeks (Fig. 3b) vernalisation treatments. Thus, model 1 successfully reproduced the essential dynamics of histone modifications and mRNA in the vernalisation treatments. Compared with these results in model 1, the simulated *AhgFLC* mRNA levels in model 2 decreased and increased faster in response to cold and warm temperatures, respectively, due to the lack of interaction between H3K27me3 and H3K4me3 at the two nucleation regions. We confirmed these differences between models 1 and 2 using independently determined parameter sets optimised for seasonal data in a natural population^24^ (Supplementary Fig. S5). Therefore, our mathematical modelling indicates that H3K27me3 buffers sudden changes in *AhgFLC* mRNA level associated with temperature changes by the interaction with H3K4me3 at the two nucleation regions. In our mathematical modelling to simulate active and repressive histone modifications at the proximal/distal nucleation regions at *AhgFLC*, we needed to analyse H3K4me3 and H3K27me3 which accumulate at these regions^24^. However, as H3K36me3 is known to be antagonistic to H3K27me3 at *AtFLC* in *A. thaliana*^15,19^, modelling of *AhgFLC* H3K36me3 dynamics should be addressed in future studies.

## Discussion

Our time series data showed that the H3K27me3 levels at all regions of *AhgFLC* were not saturated even after 8 weeks of cold, and continued to increase up to 14 weeks of cold. These results are similar to those reported for the Lov-1 accession of *A. thaliana*^18,19^; however, differences between annual and perennial plants exist in the response to warm conditions following exposure to long-term cold. In the annual Lov-1 accession of *A. thaliana*, reduction of H3K27me3 at the *AtFLC* locus is limited and the level remains high at the gene body under warm conditions following a sufficient duration of cold, which is called the perpetuated state^18,19^. In perennial *A. halleri*, high H3K27me3 levels over the entire *AhgFLC* locus were reset under warm conditions regardless of the duration of cold exposure. Furthermore, Lov-1 *AtFLC* repression is maintained up to 10 days of warm and the expression reactivates after 30 days of warm^19^, whereas *AhgFLC* expression reactivates after only 3 days of warm. This difference in the time-course of the *FLC* reactivation presumably represents the difference in their mechanisms as explained below. In Lov-1, *AtFLC* repression is unstable and susceptible to DNA replication which disrupts histone modifications and causes *AtFLC* reactivation^19^. In *A. halleri*, the influence of DNA replication on the reduction of H3K27me3 would be minimal, given the fact that we analysed *AhgFLC* H3K27me3 levels in fully expanded leaves wherein cell division and endoreduplication are inactive^35,36^. We previously revealed that *AhgFLC* transcription starts earlier than the reduction of H3K27me3 at the locus^24^, which might trigger the active removal of the modification by recruiting H3K27me3 demethylases. Similar processes have been proposed as a candidate mechanism of Lov-1 *AtFLC* reactivation^19^.

Single-nucleotide polymorphisms (SNPs) around TSS of Lov-1 *AtFLC* relate to postcold instability of the gene and requirement for longer cold than Col *FRI*^Sf2^ for gene silencing^18,19^. SNPs at *FLC* orthologues also relate to annual and perennial life cycles in *Arabis* species^29^. In *A. halleri*, temperature threshold for *AhgFLC* silencing is different between plants from different geographic areas^21^. Therefore, it should be investigated whether SNPs at *AhgFLC* may contribute to the different vernalisation responses.

Previously, we reported that H3K27me3 is required for the long-term responses of *AhgFLC* expression in a natural fluctuating environment^24^. In this study, we analysed the responses to cold and warm temperatures separately, and showed that H3K27me3 prevents a rapid reactivation of *AhgFLC* although H3K27me3 is finally reset in response to warm conditions after vernalisation. This indicates that H3K27me3 may function as a buffer against a rapid temperature increase, moderating the change speed of *AhgFLC* rather than maintaining the silenced state. Consequently, *AhgFT* expression is induced within a time window when *AhgFLC* expression is low, leading to flowering. In addition, our data revealed that the duration of cold exposure could explain the lower level of H3K27me3 at *AhgFLC* reported in laboratory samples (after 6 weeks cold) compared with field samples (in winter)^34^.

A previous study showed that *AhgFLC* expression is higher at warmer temperatures, and predicted that the flowering period decreases with climate warming based on a mathematical modelling^21^. Consistently, we showed that early *AhgFLC* reactivation due to an inadequate period of cold delays the initiation of flowering. These studies focused on the time to flower, and the influence of high *AhgFLC* expression on plant fitness should be addressed: we need to measure other reproductive traits, such as total flower numbers in future studies. The time-series analyses of reactivation of perennial *FLC* performed in this study will help reveal the molecular basis of reproductive strategies of plants with various life histories.

## Methods

### Plant materials and growth conditions

For the vernalisation experiment, we selected 24 *A*. *halleri* plants growing in a natural population at Monzen, Naka-ku, Taka-cho, Hyogo Prefecture, Japan (35° 04ʹ N, 134° 54ʹ E, altitude 140–155 m). Plants were selected along a stream in a sampling area of approximately 20 m × 230 m. The area was divided into three sections, and eight plants were selected from each section (> 2 m between plants). Four plants from each section were allocated to each of the 8 weeks and 14 weeks vernalisation treatments (in total 12 plants for each experiment). The plants were transferred from the field to the laboratory on 29 October 2013, when *AhgFLC* was highly expressed^20,23,24^. The plants were transplanted into pots and grown on outdoor pot shelves at the Center for Ecological Research, Kyoto University, Otsu, Shiga prefecture, Japan (34°58ʹ N, 135°57ʹ E, altitude 150 m). After a 10-day period of growth adjustment in the outdoor pots, the plants were grown under cold conditions at 4 °C with 12 h light/12 h dark for 8 or 14 weeks, and then grown under warm conditions at 20/15 °C with 12 h light/12 h dark. We used the same photoperiod between cold and warm conditions to measure the effect of temperature on plant phenology separately from photoperiod. The 12 h light/12 h dark condition imitates the day length in our field site in spring (March–April) when plants start reproductive growth^20,24^. The maximum and minimum day length in our field site are approximately 14 and 10 h, respectively^20^.

### Leaf sampling

Leaves were sampled at 13–17 days interval under cold conditions, and 3–7 days interval after transfer to warm conditions. On each date, sampling started at 12.00, and was completed within 1.5 h (by 13.30). For RNA samples, one small young leaf (approximately 0.01 g) was harvested from each of the 12 plants, and four biological replicates were prepared by pooling three leaves for each replicate on each sampling date. Harvested leaves were preserved in RNA*later* Stabilization Solution (Thermo Fisher Scientific, Waltham, MA, USA) on ice during sampling and stored at − 20 °C until RNA extraction. For ChIP samples, one fully expanded young leaf (approximately 0.1 g) was harvested from each of the 12 plants, and four biological replicates were prepared by pooling three leaves per replicate (0.3 g per replicate) on each sampling date. Harvested leaves were fixed with 1% formaldehyde in phosphate-buffered saline (PBS), using vacuum infiltration twice for 5 min each. The cross-linking reaction was quenched with the addition of glycine to a final concentration of 125 mM and vacuum infiltration once for 5 min. Cross-linked samples were washed with PBS, frozen in liquid nitrogen, and stored at − 80 °C until chromatin extraction.

### RNA extraction and RT-qPCR

RNA was extracted using an RNeasy Plant Mini Kit (QIAGEN, Hilden, Germany). The extracted RNA was quantified using a Qubit Fluorometer and Qubit RNA HS Assay Kits (Thermo Fisher Scientific). cDNA was synthesised using a High-Capacity cDNA Reverse Transcription Kit (Thermo Fisher Scientific). qPCR was performed in duplicate with the appropriate primers (Supplementary Table S1). A standard cDNA sample produced from non-vernalised plants was used for normalisation. *AhgFLC* mRNA levels were normalised to those of either *AhgACT2* or *AhgPP2AA3*^34^.

### ChIP-qPCR

ChIP experiments were conducted following the protocol described by Gendrel et al.^37^, with some modifications. Chromatin was extracted from 0.3 g pooled leaves using extraction buffer 1–3 by centrifugation. Extracted chromatin was sonicated eight times for 15 s each using a Q700 Sonicator (Qsonica, Newtown, CT, USA) at 10% power output. Samples were centrifuged, and the supernatant was diluted in ChIP dilution buffer (up to 900 μl). This solution was incubated with Dynabeads Protein G (Thermo Fisher Scientific) at 4 °C for 1 h with rotation for pre-clearing. The solution was then incubated with antibodies for 5 h. The antibody dilutions were as follows: 1:500 for anti-H3K27me3 (07-449; Millipore, Billerica, MA, USA) and anti-H3K4me3 (07-473; Millipore), and 1:1,000 for anti-histone H3 (ab1791; Abcam, Cambridge, UK). After incubation with the antibody, Dynabeads Protein G was added, and samples were incubated at 4 °C for an additional 2 h. After immunoprecipitation, the beads were washed sequentially with low salt, high salt, LiCl, and TE buffers. The chromatin was then eluted with elution buffer, cross-linking was reversed by heating at 65 °C for 12 h, and proteinase K treatment was conducted at 45 °C for 1 h. DNA was extracted using phenol/chloroform, ethanol-precipitated, and then resuspended in 50 μl of TE buffer. qPCR was performed in duplicate by using the appropriate primers (Supplementary Table S1). To collect data, the 7,300 System SDS Software v1.3 was used. We analysed six regions along the *AhgFLC* locus: amplicons I–VI. Amplicons I and II were selected within the previously defined nucleation region^14−16,29^; amplicons III–V were selected within the gene body with a distance of more than 1 kbp between them; amplicons VI were selected around the 3′ end of the locus. The absolute amount of H3K4me3/H3K27me3 ChIP DNA, expressed as % of input, was divided by the absolute amount of H3 ChIP DNA, expressed as % of input at the same region. In addition, *AhgSTM* and *AhgFUS3* were used for H3K27me3 ChIP, and *AhgACT2* and *AhgPP2AA3* were used for H3K4me3 ChIP as internal controls^34^. Both *AhgFLC* H3K27me3 and H3K4me3 levels are presented on a linear scale.

### Reproductive transition

On each date of leaf sampling, we recorded whether each of the 12 plants had reached the three sequential reproductive stages (bolting, flowering, and reversion), in both the 8 weeks and 14 weeks vernalisation treatments. Bolting was defined as the stage when flowering stems were longer than 3 mm before presenting open flowers. Flowering was defined as the stage when the flowers had opened. Reversion was defined as the stage when leaves had formed at the top of flowering stems at the end of the flowering period. During reversion, plants form aerial rosettes and generate roots at the top of flowering stems and most of the lateral meristems (nodes of cauline leaves), facilitating the establishment of vegetative (clonal) offspring.

### Full mathematical model with H3K27me3

For the full model (model 1 with H3K27me3), the mathematical model established by Nishio et al.^24^ was used. In this model, H3K27me3 at the *AhgFLC* locus was classified into four states: U_N_U_D_ (neither nucleation regions are modified), M_N_U_D_ (only the nucleation region is modified), M_N_M_D_ (both nucleation regions are modified), and U_N_M_D_ (only the distal nucleation region is modified)—the proportions: *u*_N_*u*_D_, *m*_N_*u*_D_, *m*_N_*m*_D_, and *u*_N_*m*_D_, respectively. The transition of H3K27me3 at the locus was assumed to be unidirectional, that is, U_N_U_D_ → M_N_U_D_ → M_N_M_D_ → U_N_M_D_ → U_N_U_D_. The H3K4me3 states were described by U_N_ and A_N_ for the nucleation region (the proportions: *u*_N_ and *a*_N_), and U_D_ and A_D_ for the distal nucleation region (the proportions: *u*_D_ and *a*_D_).

The transitions U_N_U_D_ → M_N_U_D_ and M_N_U_D_ → M_N_M_D_ were assumed to be induced by cold and warm temperatures, represented by the functions of temperature, *μ*(*T*) and *ν*(*T*), respectively. These functions are given by the logistic equations of the form:

1 $$\mu \left( T \right) = \frac{\zeta }{{1 + \exp \left( {\alpha \left( {T - \theta_{1} } \right)} \right)}} ,$$

2 $$\nu \left( T \right) = \frac{\eta }{{1 + \exp \left( { - \beta \left( {T - \theta_{2} } \right)} \right)}} .$$

The M_N_M_D_ → U_N_M_D_ transition was assumed to be induced by H3K4me3 at the nucleation region (*a*_N_), and the U_N_M_D_ → U_N_U_D_ transition was assumed to occur at a constant rate (*λ*). Thus, the time derivatives of the H3K27me3 states at the *AhgFLC* locus are given by

3 $$\frac{{du_{{\text{N}}} u_{{\text{D}}} }}{dt} = \lambda u_{{\text{N}}} m_{{\text{D}}} - \mu \left( T \right) u_{{\text{N}}} u_{{\text{D}}} ,$$

4 $$\frac{{dm_{{\text{N}}} u_{{\text{D}}} }}{dt} = \mu \left( T \right) u_{{\text{N}}} u_{{\text{D}}} - \nu \left( T \right) m_{{\text{N}}} u_{{\text{D}}} ,$$

5 $$\frac{{dm_{{\text{N}}} m_{{\text{D}}} }}{dt} = \nu \left( T \right) m_{{\text{N}}} u_{{\text{D}}} - \kappa a_{{\text{N}}} m_{{\text{N}}} m_{{\text{D}}} ,$$

6 $$\frac{{du_{{\text{N}}} m_{{\text{D}}} }}{dt} = \kappa a_{{\text{N}}} m_{{\text{N}}} m_{{\text{D}}} - \lambda u_{{\text{N}}} m_{{\text{D}}} .$$

H3K4me3 at the nucleation region and the distal nucleation region were assumed to accumulate in response to warm [*ξ*(*T*)] and cold [*τ*(*T*)] temperatures, respectively. These functions are given by the logistic equations of the form:

7 $$\xi \left( T \right) = \frac{\iota }{{1 + \exp \left( { - \gamma \left( {T - \theta_{3} } \right)} \right)}} ,$$

8 $$\tau \left( T \right) = \frac{\rho }{{1 + \exp \left( {\varepsilon \left( {T - \theta_{4} } \right)} \right)}} .$$

H3K4me3 at the distal nucleation region was assumed to negatively affect that at the nucleation region, and vice versa. H3K27me3 was also assumed to negatively affect H3K4me3 at the two nucleation regions. Thus, the time derivatives of the H3K4me3 states at the *AhgFLC* locus are given by

9 $$\frac{{da_{{\text{N}}} }}{dt} = \xi \left( T \right)\left( {1 - a_{{\text{D}}} } \right) u_{{\text{N}}} - \varphi \left( {m_{{\text{N}}} u_{{\text{D}}} + m_{{\text{N}}} m_{{\text{D}}} } \right) a_{{\text{N}}} ,$$

10 $$\frac{{da_{{\text{D}}} }}{dt} = \tau \left( T \right)\left( {1 - a_{{\text{N}}} } \right) u_{{\text{D}}} - \psi \left( {m_{{\text{N}}} m_{{\text{D}}} + u_{{\text{N}}} m_{{\text{D}}} } \right) a_{{\text{D}}} .$$

The observed histone modification levels at the nucleation region and the distal nucleation region were represented by the mean level of amplicons I and II, and the level of amplicon VI, respectively. To represent the simulated histone modification levels in each vernalisation treatment, *m*_N_*u*_D_ + *m*_N_*m*_D_ and *m*_N_*m*_D_ + *u*_N_*m*_D_ were multiplied by the maximum observed levels of H3K27me3 at the nucleation region and the distal nucleation region, respectively; *a*_N_ and *a*_D_ were multiplied by those of H3K4me3 at these regions, respectively.

Simulations were performed using the ode function of the deSolve package of R v3.3.2. Model parameters were optimised by minimising the residual sum of squares between the model output and the observed data in both vernalisation treatments in two steps. First, we randomly selected 1000 parameter sets from the uniform distribution, and selected the parameter set that best fitted the observed data. Using the selected parameter set as the initial values, the parameters were then optimised by the simulated annealing (SANN) method^38^ using the optim function of R. Explanations and the determined values of the parameters are listed in Supplementary Table S2. For the simulation, we used temperature regimes in growth chambers (4 °C in cold and 20/15 °C D/N in warm).

We modelled the observed *AhgFLC* mRNA level by linear regression with the observed H3K4me3 level at the nucleation region given by

11 $$log_{10} \left( {RNA} \right) = \sigma log_{10} \left( {K4 \,at\, NR} \right) + \omega .$$

Using the determined regression coefficient and intercept, we reproduced the simulated *AhgFLC* mRNA level from the simulated H3K4me3 level at the nucleation region. The simulated mRNA level was standardised to ensure that the amplitude was even between models.

### Mathematical model without H3K27me3

For the model that lacks H3K27me3 (model 2 without H3K27me3), all variables and parameters regarding the H3K27me3 states at the *AhgFLC* locus were excluded from the model. The time derivatives of the H3K4me3 states at the *AhgFLC* locus are given by

12 $$\frac{{da_{{\text{N}}} }}{dt} = \xi \left( T \right)\left( {1 - a_{{\text{D}}} } \right) u_{{\text{N}}} - \varphi a_{{\text{N}}} ,$$

13 $$\frac{{da_{{\text{D}}} }}{dt} = \tau \left( T \right)\left( {1 - a_{{\text{N}}} } \right) u_{{\text{D}}} - \psi a_{{\text{D}}} .$$

Using the regression coefficient and intercept in the linear regression represented by Eq. (11), we reproduced the simulated *AhgFLC* mRNA level from the simulated H3K4me3 level at the nucleation region.

## Supplementary information

Supplementary file 1.
