# Supplementary material for: Duration of cold exposure defines the rate of reactivation of a perennial FLC orthologue via H3K27me3 accumulation
Source: Sci Rep. 2020 Sep 29;10:16056. doi: 10.1038/s41598-020-72566-7 (PMC7525499; doi:10.1038/s41598-020-72566-7)
Supplement: Supplementary file 1 [file 41598_2020_72566_MOESM1_ESM.pdf]

Supplementary Infaormation for

Duration of cold exposure defines the rate of reactivation of a perennial *FLC* orthologue via H3K27me3 accumulation

Haruki Nishio*, Koji Iwayama, and Hiroshi Kudoh*

*Corresponding authors: Haruki Nishio (harukin218@gmail.com) or

Hiroshi Kudoh (kudoh@ecology.kyoto-u.ac.jp).

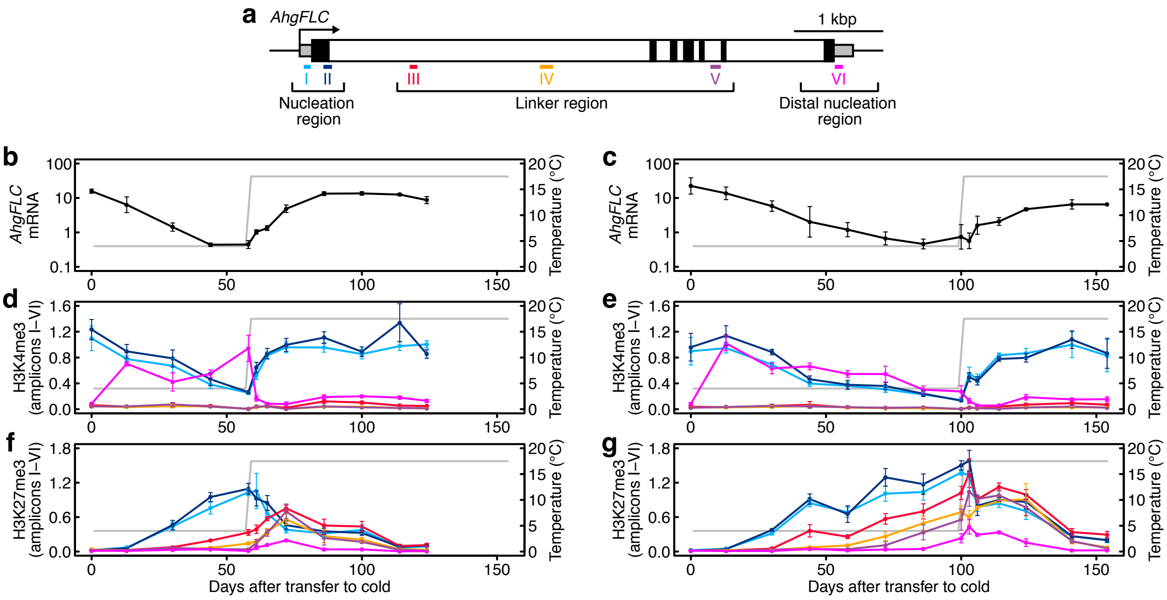

Supplementary Figure S1. Dynamics of *AhgFLC* mRNA and histone modification levels in the vernalisation treatments, normalised by alternative reference genes. (**a**) Structure of the *AhgFLC* locus with untranslated regions (grey), exons (black), and introns (white); distribution of six H3K4me3 and H3K27me3 ChIP amplicons in different colours and the definitions of the nucleation, linker, and distal nucleation region. (**b**–**g**) The dynamics of *AhgFLC* mRNA (**b**, **c**), H3K4me3 at amplicons I–VI (**d**, **e**), and H3K27me3 at amplicons I–VI (**f**, **g**) in the 8 wk (**b**, **d**, **f**) and 14 wk (**c**, **e**, **g**) vernalisation treatments. In (**b**–**g**), temperature regimes are represented by grey lines (4°C in cold and 20/15°C D/N in warm, shown by the average value). The colour code in (**d**–**g**) corresponds to that in (**a**). In (**b**–**g**), the means and standard errors of biological replicates are shown. n = 2–4 (average, 3.3) and 1–4 (average, 3.4) for mRNA in the 8wk and 14 wk vernalisation treatments, respectively. n = 4 for H3K4me3 and H3K27me3 at all amplicons in both vernalisation treatments. For each replicate, a pool of leaves from three plants (out of 12 plants) was analysed. The qPCR data are represented relative to *AhgPP2AA3* (mRNA and H3K4me3) and *AhgFUS3* (H3K27me3).

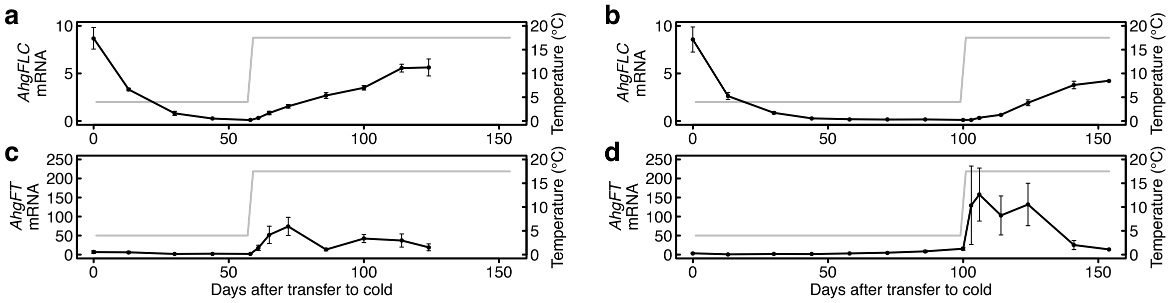

Supplementary Figure S2. Dynamics of *AhgFLC* and *AhgFT* mRNA levels in the vernalisation treatments. (**a**–**d**) The dynamics of *AhgFLC* mRNA (**a**, **b**) and *AhgFT* mRNA (**c**, **d**) in the 8 wk (**a**, **c**) and 14 wk (**b**, **d**) vernalisation treatments. Temperature regimes are represented by grey lines (4°C in cold and 20/15°C D/N in warm conditions, shown by the average value). The means and standard errors of biological replicates are shown. n = 2−4 (average, 3.3) and 1−4 (average, 3.4) for mRNA in the 8wk and 14 wk vernalisation treatments, respectively. For each replicate, a pool of leaves from three plants (out of 12 plants) was analysed. The qPCR data are represented relative to *AhgACT2*.

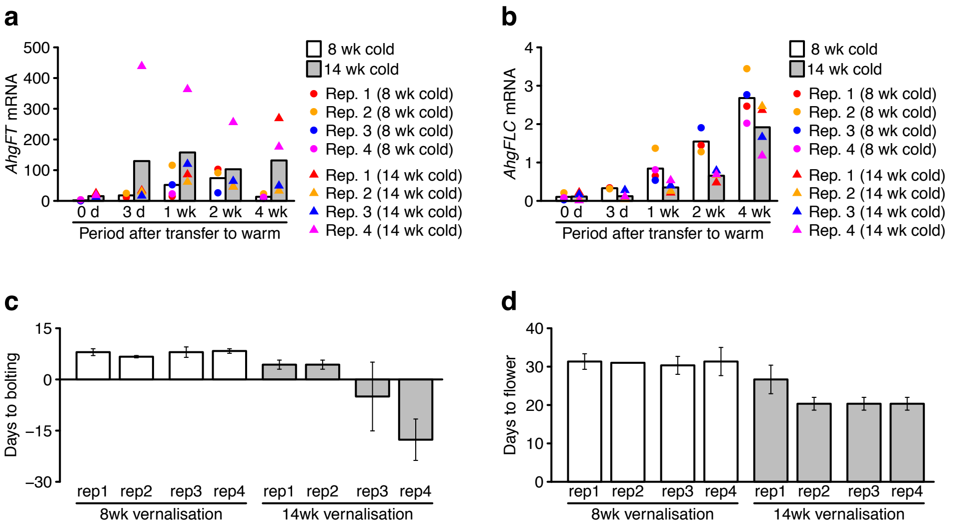

Supplementary Figure S3. The expression of flowering genes and time to reproductive transition after vernalisation in each replicate. (**a**, **b**) Effects of cold duration (8 and 14 wk) and period after transfer to warm conditions on the expression of *AhgFT* (**a**) and *AhgFLC* (**b**) mRNA. The mRNA level in each biological replicate is represented by a distinct symbol. Numbers of replicates are 2−4 (average, 3.4) and 4 for mRNA in the 8 wk and 14 wk vernalisation treatments, respectively. For each replicate, a pool of leaves from three plants (out of 12 plants) were analysed. The qPCR data are represented relative to *AhgACT2*. (**c**, **d**) Days to bolting (**c**) and to flower (**d**) under warm conditions after 8 and 14 wk vernalisation. Data are shown for each replicate used to measure the mRNA levels. The means and standard errors are shown. Numbers of plants within each replicate is three for bolting and flowering time in both vernalisation experiments. The minus values in (**c**) indicate that bolting has started during vernalisation. In (**a**−**d**), the same replicate number represents the same set of plants in each of 8 and 14 wk vernalisation treatments.

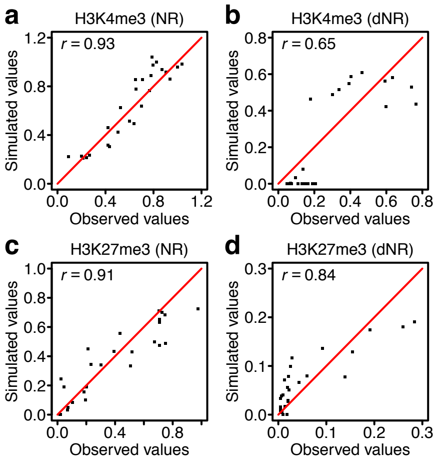

Supplementary Figure S4. Fitting of the simulated values of histone modifications at *AhgFLC* to the observed values in mathematical modelling. (**a**–**d**) Correlation between the observed and simulated values for *AhgFLC* H3K4me3 levels at the nucleation region (NR, **a**) and the distal nucleation region (dNR, **b**) and *AhgFLC* H3K27me3 levels at NR (**c**) and dNR (**d**). Both values in the 8 wk and 14 wk vernalisation treatments were included in each plot. The observed values are shown as the means of biological replicates. Spearman's correlation coefficient (*r*) is shown in each plot.

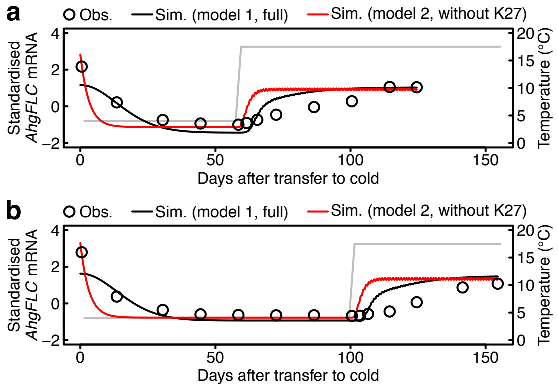

Supplementary Figure S5. Comparison of *AhgFLC* mRNA dynamics between the mathematical models with and without the effect of H3K27me3 using the parameter set optimised for seasonal data in a natural population[1]. (**a**) Simulated *AhgFLC* mRNA levels in the 8 wk vernalisation treatment are compared between models with (black line) and without (red line) H3K27me3, and are shown with the observed values (open circles). (**b**) Simulated *AhgFLC* mRNA levels in the 14 wk vernalisation treatment. Otherwise the same as in (**a**). The observed values are shown as the means of biological replicates. Temperature regimes are represented by grey lines (4°C in cold and 20/15°C D/N in warm, shown by the average value).

Gene	Experiment	Primer sequence
AhgFLC amplicon I	ChIP-qPCR	5′-AAAAGGAAAGAGAAGAACGCTTAG-3′
		5′-AGAGGGCTTTATTCCCTAGTTTG-3′
AhgFLC amplicon II	ChIP-qPCR	5′-GTCGCTCTTCTCGTCGTCTC-3′
		5′-GGGAAATAAATGGAAACCCAGA-3′
AhgFLC amplicon III	ChIP-qPCR	5′-GAAAACCGACAATCACACAACC-3′
		5′-TAGAGGCACCAAAGAAACAAGG-3′
AhgFLC amplicon IV	ChIP-qPCR	5′-ATGTCATCACTTTGTGGCTCATC-3′
		5′-CATGTAAACGCAGCCGTAATC-3′
AhgFLC amplicon V	ChIP-qPCR	5′-TCTTAACGAGCTTGCACACATC-3′
		5′-AAGACCGACACTTTCAGCAAATAC-3′
AhgFLC amplicon VI	ChIP-qPCR	5′-CGGCGGTTAAAATCAAAATC-3′
		5′-CAAACGCTCGCCCTTATC-3′
AhgSTM	ChIP-qPCR	5′-ATCAGTCACACCACCAACAAAGTAG-3′
		5′-AGAGAATAAGCAGGGGCACAAG-3′
AhgACT2	ChIP-qPCR	5′-GCGACCAGACAGAGAAAGAAGG-3′
		5′-GATGGAGAAAAGCGGAAGAAGA-3′
AhgFUS3	ChIP-qPCR	5′-ATTCTCAACGGAGCCCAAAC-3′
		5′-AACCTCCAACGACACTCCTCTC-3′
AhgPP2AA3	ChIP-qPCR	5′-CAGCGTAATCGGTAAAATCTCG-3′
		5′-CCGTCCCAATCTAGAGAGAAACTAC-3′
AhgFLC	RT-qPCR	5′-CGGCGGTTAAAATCAAAATC-3′
		5′-CAAACGCTCGCCCTTATC-3′
AhgFT	RT-qPCR	5′-TGGTGGATCCAGATGTTCCTAGT-3′
		5′-TCACCAACCAATGGAGATATTCTC-3′
AhgACT2	RT-qPCR	5′-TCCCTCAGCACATTCCAGCAGAT-3′
		5′-AACGATTCCTGGACCTGCCTCATC-3′
AhgPP2AA3	RT-qPCR	5′-GTATGCACATGTTTTGCTTCCAC-3′
		5′-CAACCAAGTCATTCTCCCTCATC-3′

Supplementary Table S1. List of the primers used for qPCR.

Symbol	Description	Optimised values in the model 1 (full model)	Optimised values in the model 2 (without H3K27me3)
$u_N u_D$	Proportion of the cells in which both two nucleation regions are not modified with H3K27me3		
$m_N u_D$	Proportion of the cells in which only the nucleation region is modified with H3K27me3		
$m_N m_D$	Proportion of the cells in which both two nucleation regions are modified with H3K27me3		
$u_N m_D$	Proportion of the cells in which only the distal nucleation region is modified with H3K27me3		
u_N, a_N	Proportion of the cells in which the nucleation region is not modified and is modified with H3K4me3, respectively		
u_D, a_D	Proportion of the cells in which the distal nucleation region is not modified and is modified with H3K4me3, respectively		
t	Time		
T	Temperature		
$\mu(T)$	Cold-dependent transition of $U_N U_D$-to-$M_N U_D$		
$v(T)$	Warm-dependent transition of $M_N U_D$-to-$M_N M_D$		
$\xi(T)$	Warm-dependent transition of U_N-to-A_N		
$\tau(T)$	Cold-dependent transition of U_D-to-A_D		
θ_1	Threshold temperature of $\mu(T)$	5.00	
θ_2	Threshold temperature of $v(T)$	10.0	
θ_3	Threshold temperature of $\xi(T)$	15.0	15.0
θ_4	Threshold temperature of $\tau(T)$	5.00	5.00
α	Slope of $\mu(T)$	10.5	
β	Slope of $v(T)$	0.410	
γ	Slope of $\xi(T)$	0.147	0.185
ε	Slope of $\tau(T)$	16.7	7.08
ζ	Maximum value of $\mu(T)$	1.38	
η	Maximum value of $v(T)$	16.0	
ι	Maximum value of ξ(T)	20.3	18.6
ρ	Maximum value of $\tau(T)$	87.7	99.3
κ	Dependence on H3K4me3 in the demethylation of H3K27me3 at the nucleation region	3.09	
λ	Demethylation rate of H3K27me3 at the distal nucleation region	6.96	
φ	Dependence on H3K27me3 in the demethylation of H3K4me3 at the nucleation region	6.34	1.93
ψ	Dependence on H3K27me3 in the demethylation of H3K4me3 at the distal nucleation region	97.7	26.4
σ	Coefficient of the linear regression to model mRNA	2.32	2.32
ω	Intercept of the linear regression to model mRNA	−1.48	−1.48

Supplementary Table S2. List of the symbols used in the simulation and optimised parameter values.

References

1. Nishio, H. et al. Repressive chromatin modification underpins the long-term expression trend of a perennial flowering gene in nature. *Nat. Commun.* **11**, 2065 (2020).
